# *catena*-Poly[[bis­(μ-2-formyl-6-meth­oxy­phenolato-1:2κ^4^ *O* ^1^,*O* ^6^:*O* ^1^,*O* ^2^)copper(II)sodium]-μ-tetra­fluorido­borate-1:1′κ^2^ *F*:*F*′]

**DOI:** 10.1107/S1600536811051713

**Published:** 2011-12-10

**Authors:** Yu Yang, Po Gao, Jing-Lin Yang, Hai-Ge Hou, Ting Gao

**Affiliations:** a School of Chemistry and Materials Science, Heilongjiang University, Harbin 150080, People’s Republic of China

## Abstract

In the title heterodinuclear complex, [CuNa(BF_4_)(C_8_H_7_O_3_)_2_]_*n*_, the Cu^II^ ion is four-coordinated by four O atoms of two 2-formyl-6-meth­oxy­phenolate ligands, giving rise to a square-planar geometry. The Na^+^ ion is six-coordinated by four O atoms from the two ligands and two F atoms of two tetra­fluoridoborate anions. The tetra­fluoridoborate anion links the Na^+^ ions, forming a one-dimensional structure along [001]. Three F atoms of the tetra­fluoridoborate anion are disordered over two sets of sites, with an occupancy ratio of 0.790 (11):0.210 (11).

## Related literature

For related heterodinuclear complexes, see: Gao *et al.* (2011 ▶); Kajiwara *et al.* (2008 ▶).
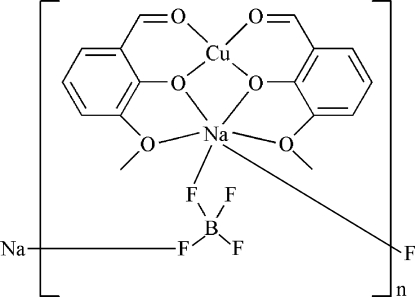


## Experimental

### 

#### Crystal data

[CuNa(BF_4_)(C_8_H_7_O_3_)_2_]
*M* *_r_* = 475.62
Monoclinic, 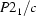

*a* = 9.932 (2) Å
*b* = 19.349 (4) Å
*c* = 9.940 (2) Å
β = 105.16 (3)°
*V* = 1843.7 (7) Å^3^
*Z* = 4
Mo *K*α radiation
μ = 1.28 mm^−1^
*T* = 293 K
0.21 × 0.18 × 0.16 mm

#### Data collection

Rigaku R-AXIS RAPID diffractometer
Absorption correction: multi-scan (*ABSCOR*; Higashi, 1995 ▶) *T*_min_ = 0.779, *T*_max_ = 0.820
17729 measured reflections
4201 independent reflections
2278 reflections with *I* > 2σ(*I*)
*R*_int_ = 0.100

#### Refinement

*R*[*F* ^2^ > 2σ(*F* ^2^)] = 0.061
*wR*(*F* ^2^) = 0.143
*S* = 1.04
4201 reflections
292 parameters
30 restraints
H-atom parameters constrained
Δρ_max_ = 0.50 e Å^−3^
Δρ_min_ = −0.34 e Å^−3^

### 

Data collection: *RAPID-AUTO* (Rigaku, 1998 ▶); cell refinement: *RAPID-AUTO*; data reduction: *CrystalStructure* (Rigaku/MSC, 2002 ▶); program(s) used to solve structure: *SHELXS97* (Sheldrick, 2008 ▶); program(s) used to refine structure: *SHELXL97* (Sheldrick, 2008 ▶); molecular graphics: *DIAMOND* (Brandenburg, 1999 ▶); software used to prepare material for publication: *SHELXTL* (Sheldrick, 2008 ▶).

## Supplementary Material

Crystal structure: contains datablock(s) global, I. DOI: 10.1107/S1600536811051713/hy2490sup1.cif

Structure factors: contains datablock(s) I. DOI: 10.1107/S1600536811051713/hy2490Isup2.hkl

Additional supplementary materials:  crystallographic information; 3D view; checkCIF report

## Figures and Tables

**Table 1:** Selected bond lengths (Å)

Cu1—O2	1.888 (3)
Cu1—O3	1.929 (3)
Cu1—O5	1.889 (3)
Cu1—O6	1.936 (3)
Na1—O1	2.615 (3)
Na1—O2	2.377 (3)
Na1—O4	2.614 (4)
Na1—O5	2.370 (3)
Na1—F1	2.215 (7)
Na1—F3′	2.344 (17)
Na1—F4^i^	2.243 (4)

Symmetry code: (i) 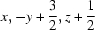.

**Fig. 1.**
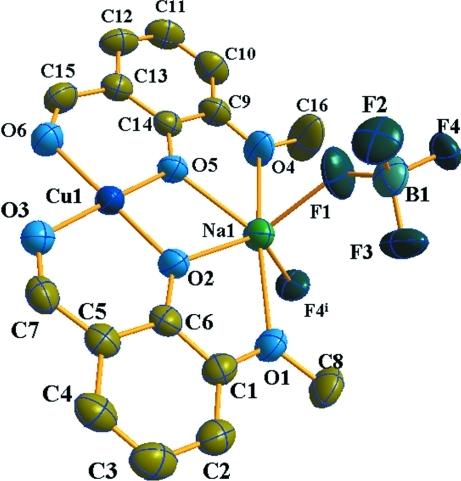
The molecular structure of the title compound, showing the 30% probability displacement ellipsoids. [Symmetry code: (i) x, 3/2-y, 1/2+z.]

## supplementary crystallographic information

### Comment

Orthovanillin is a commercial ligand that can yeild heterodinuclear 3d-4f complexes with two different coordination sites; 3d ions have a marked affinity for the inner O_2_O_2_ site, whereas 4f ions prefer the larger outer O_2_O_2_ coordination site (Kajiwara *et al.*, 2008). Recently, we were interested in the nature of the products obtained by reacting a 3d complex with alkali metal ions (Gao *et al.*, 2011). In this paper we reacted a Cu complex with sodium tetrafluoridoborate to yield a heterodinuclear complex. As shown in Fig. 1, the Cu^II^ ion is four-coordinated by two aldehyde O atoms and two phenolate O atoms from two orthovanillin ligands (Table 1). The Cu^II^ ion is inserted to inner cavity in a square-planar geometry. The Na^+^ ion is ligated by two phenolate O atoms, two methoxyl O atoms and two F atoms from two tetrafluoridoborate anions. The Cu^II^ and Na^+^ ions are bridged by the phenolate O atoms.

### Experimental

To a solution of *o*-vanillin (0.046 g, 0.20 mmol) in dichloromethane (5 ml) was added to a solution of copper(II) acetate monohydrate (0.040 g, 0.20 mmol) and sodium tetrafluoridoborate (0.034 g, 0.20 mmol) in ethanol (5 ml). The mixture was stirred, heated under reflux (30 min) and then allowed to cool to room temperature (yield: 70%). The crystals suitable for X-ray determination were obtained by slow diffusion of diethylether into the solution for one week. Analysis, calculated for C_16_H_14_BCuF_4_NaO_6_: C 40.40, H 2.97%; found: C 40.38, H 2.98%.

### Refinement

Three F atoms were disordered each in two positions with an occupancy factors of 0.790 (11) for F1, F2, F3 and 0.210 (11) for F1', F2', F3'. H atoms bound to C atoms were placed in calculated positions and treated as riding on their parent atoms, with C—H = 0.93 (aromatic and aldehyde) and 0.96 (methyl) Å and with *U*_iso_(H) = 1.2(1.5 for methyl)*U*_eq_(C).

### Crystal data

**Table:**

[CuNa(BF_4_)(C_8_H_7_O_3_)_2_]	*F*(000) = 956
*M**_r_* = 475.62	*D*_x_ = 1.714 Mg m^−^^3^
Monoclinic, *P*2_1_/*c*	Mo *K*α radiation, λ = 0.71073 Å
Hall symbol: -P 2ybc	Cell parameters from 9827 reflections
*a* = 9.932 (2) Å	θ = 3.0–27.5°
*b* = 19.349 (4) Å	µ = 1.28 mm^−^^1^
*c* = 9.940 (2) Å	*T* = 293 K
β = 105.16 (3)°	Block, colorless
*V* = 1843.7 (7) Å^3^	0.21 × 0.18 × 0.16 mm
*Z* = 4	

### Data collection

**Table:**

Rigaku R-AXIS RAPID diffractometer	4201 independent reflections
Radiation source: fine-focus sealed tube	2278 reflections with *I* > 2σ(*I*)
graphite	*R*_int_ = 0.100
ω scans	θ_max_ = 27.5°, θ_min_ = 3.0°
Absorption correction: multi-scan (*ABSCOR*; Higashi, 1995)	*h* = −12→12
*T*_min_ = 0.779, *T*_max_ = 0.820	*k* = −25→25
17729 measured reflections	*l* = −11→12

### Refinement

**Table:**

Refinement on *F*^2^	Primary atom site location: structure-invariant direct methods
Least-squares matrix: full	Secondary atom site location: difference Fourier map
*R*[*F*^2^ > 2σ(*F*^2^)] = 0.061	Hydrogen site location: inferred from neighbouring sites
*wR*(*F*^2^) = 0.143	H-atom parameters constrained
*S* = 1.04	*w* = 1/[σ^2^(*F*_o_^2^) + (0.0582*P*)^2^] where *P* = (*F*_o_^2^ + 2*F*_c_^2^)/3
4201 reflections	(Δ/σ)_max_ < 0.001
292 parameters	Δρ_max_ = 0.50 e Å^−^^3^
30 restraints	Δρ_min_ = −0.34 e Å^−^^3^

### Fractional atomic coordinates and isotropic or equivalent isotropic displacement parameters (Å^2^)

**Table:**

	*x*	*y*	*z*	*U*_iso_*/*U*_eq_	Occ. (<1)
F1'	0.821 (3)	0.8431 (15)	0.453 (4)	0.135 (10)	0.210 (11)
F2'	0.979 (4)	0.8027 (19)	0.428 (4)	0.174 (14)	0.210 (11)
F3'	0.874 (2)	0.7798 (9)	0.6030 (15)	0.075 (6)	0.210 (11)
F1	0.7789 (9)	0.8066 (5)	0.5476 (9)	0.144 (3)	0.790 (11)
F2	0.8679 (9)	0.8364 (4)	0.3784 (7)	0.132 (3)	0.790 (11)
F3	0.9995 (6)	0.7795 (3)	0.5612 (7)	0.131 (3)	0.790 (11)
F4	0.8288 (4)	0.72587 (18)	0.4087 (4)	0.1037 (13)	
B1	0.8662 (8)	0.7866 (3)	0.4740 (7)	0.0628 (17)	
C1	1.1275 (4)	0.9403 (3)	0.8798 (5)	0.0456 (11)	
C2	1.2649 (5)	0.9573 (3)	0.9383 (5)	0.0550 (13)	
H2	1.3310	0.9224	0.9656	0.066*	
C3	1.3060 (5)	1.0264 (3)	0.9568 (5)	0.0615 (14)	
H3	1.3991	1.0372	0.9971	0.074*	
C4	1.2118 (5)	1.0780 (3)	0.9166 (5)	0.0549 (13)	
H4	1.2412	1.1238	0.9266	0.066*	
C5	1.0682 (4)	1.0625 (2)	0.8591 (4)	0.0427 (11)	
C6	1.0246 (4)	0.9926 (2)	0.8378 (4)	0.0402 (10)	
C7	0.9744 (5)	1.1182 (3)	0.8252 (4)	0.0503 (12)	
H7	1.0138	1.1621	0.8362	0.060*	
C8	1.1712 (5)	0.8188 (3)	0.8887 (6)	0.0687 (15)	
H8A	1.2427	0.8250	0.8409	0.103*	
H8B	1.1231	0.7761	0.8600	0.103*	
H8C	1.2127	0.8178	0.9875	0.103*	
C9	0.4449 (5)	0.8729 (3)	0.6389 (5)	0.0559 (13)	
C10	0.3042 (5)	0.8629 (3)	0.5859 (5)	0.0650 (15)	
H10	0.2700	0.8181	0.5687	0.078*	
C11	0.2110 (5)	0.9186 (3)	0.5569 (5)	0.0659 (16)	
H11	0.1160	0.9107	0.5212	0.079*	
C12	0.2593 (5)	0.9832 (3)	0.5809 (5)	0.0621 (15)	
H12	0.1971	1.0201	0.5627	0.075*	
C13	0.4038 (5)	0.9963 (3)	0.6338 (4)	0.0477 (12)	
C14	0.4998 (4)	0.9407 (2)	0.6613 (4)	0.0435 (11)	
C15	0.4493 (5)	1.0659 (3)	0.6616 (5)	0.0598 (14)	
H15	0.3793	1.0992	0.6435	0.072*	
C16	0.5011 (7)	0.7525 (3)	0.6450 (9)	0.120 (3)	
H16A	0.4328	0.7411	0.6941	0.180*	
H16B	0.5804	0.7225	0.6753	0.180*	
H16C	0.4614	0.7465	0.5467	0.180*	
Cu1	0.73679 (5)	1.03129 (3)	0.74231 (6)	0.0460 (2)	
Na1	0.80631 (19)	0.85912 (9)	0.75096 (19)	0.0555 (5)	
O1	1.0749 (3)	0.87466 (17)	0.8560 (3)	0.0542 (8)	
O2	0.8957 (3)	0.97361 (15)	0.7830 (3)	0.0468 (8)	
O3	0.8430 (3)	1.11575 (16)	0.7817 (3)	0.0538 (8)	
O4	0.5441 (3)	0.82251 (18)	0.6728 (4)	0.0732 (11)	
O5	0.6356 (3)	0.94771 (15)	0.7049 (3)	0.0482 (8)	
O6	0.5707 (3)	1.08808 (17)	0.7071 (3)	0.0589 (9)	

### Atomic displacement parameters (Å^2^)

**Table:**

	*U*^11^	*U*^22^	*U*^33^	*U*^12^	*U*^13^	*U*^23^
F1'	0.144 (13)	0.108 (12)	0.148 (14)	0.046 (9)	0.031 (9)	0.023 (9)
F2'	0.165 (16)	0.181 (17)	0.184 (17)	−0.004 (10)	0.061 (10)	−0.001 (10)
F3'	0.108 (11)	0.074 (9)	0.043 (7)	0.021 (8)	0.021 (7)	−0.014 (6)
F1	0.160 (6)	0.160 (6)	0.149 (6)	−0.009 (5)	0.106 (5)	−0.052 (5)
F2	0.175 (6)	0.118 (5)	0.116 (4)	0.009 (4)	0.058 (4)	0.059 (4)
F3	0.110 (5)	0.084 (4)	0.151 (6)	−0.006 (3)	−0.051 (4)	0.006 (3)
F4	0.133 (3)	0.070 (3)	0.095 (3)	−0.002 (2)	0.008 (2)	−0.034 (2)
B1	0.083 (4)	0.038 (3)	0.068 (4)	0.008 (3)	0.020 (4)	0.009 (3)
C1	0.044 (2)	0.049 (3)	0.044 (2)	0.003 (2)	0.010 (2)	0.006 (2)
C2	0.047 (3)	0.063 (4)	0.053 (3)	0.006 (2)	0.010 (2)	0.003 (3)
C3	0.041 (2)	0.082 (4)	0.061 (3)	−0.009 (3)	0.014 (2)	−0.006 (3)
C4	0.050 (3)	0.063 (4)	0.052 (3)	−0.018 (3)	0.015 (2)	−0.010 (3)
C5	0.045 (2)	0.046 (3)	0.037 (2)	−0.006 (2)	0.013 (2)	−0.001 (2)
C6	0.043 (2)	0.042 (3)	0.035 (2)	−0.007 (2)	0.0077 (19)	−0.0005 (19)
C7	0.063 (3)	0.039 (3)	0.048 (3)	−0.008 (2)	0.012 (2)	−0.005 (2)
C8	0.073 (3)	0.052 (3)	0.079 (4)	0.022 (3)	0.017 (3)	0.008 (3)
C9	0.047 (3)	0.062 (4)	0.055 (3)	−0.011 (2)	0.007 (2)	−0.006 (3)
C10	0.055 (3)	0.074 (4)	0.069 (3)	−0.018 (3)	0.021 (3)	−0.012 (3)
C11	0.042 (3)	0.095 (5)	0.060 (3)	−0.014 (3)	0.012 (2)	0.000 (3)
C12	0.041 (3)	0.089 (5)	0.054 (3)	0.007 (3)	0.008 (2)	0.010 (3)
C13	0.046 (2)	0.055 (3)	0.040 (2)	−0.003 (2)	0.009 (2)	−0.001 (2)
C14	0.043 (2)	0.050 (3)	0.036 (2)	−0.002 (2)	0.007 (2)	−0.005 (2)
C15	0.049 (3)	0.064 (4)	0.062 (3)	0.014 (3)	0.007 (2)	0.002 (3)
C16	0.079 (4)	0.054 (4)	0.212 (8)	−0.016 (3)	0.010 (5)	−0.023 (5)
Cu1	0.0427 (3)	0.0384 (3)	0.0514 (3)	−0.0002 (3)	0.0023 (2)	−0.0014 (3)
Na1	0.0595 (10)	0.0392 (11)	0.0616 (11)	−0.0008 (9)	0.0046 (9)	0.0018 (9)
O1	0.0483 (17)	0.041 (2)	0.068 (2)	0.0085 (15)	0.0063 (15)	0.0036 (16)
O2	0.0418 (15)	0.0359 (17)	0.0562 (18)	−0.0042 (14)	0.0011 (14)	−0.0001 (15)
O3	0.0541 (19)	0.042 (2)	0.058 (2)	−0.0009 (15)	0.0031 (16)	0.0009 (15)
O4	0.055 (2)	0.045 (2)	0.110 (3)	−0.0091 (17)	0.0063 (19)	−0.014 (2)
O5	0.0379 (15)	0.0449 (19)	0.0561 (19)	−0.0041 (13)	0.0019 (14)	−0.0034 (15)
O6	0.0485 (18)	0.049 (2)	0.074 (2)	0.0037 (16)	0.0064 (17)	−0.0013 (18)

### Geometric parameters (Å, °)

**Table:**

F1'—B1	1.18 (3)	C10—C11	1.400 (7)
F2'—B1	1.35 (3)	C10—H10	0.9300
F3'—B1	1.270 (16)	C11—C12	1.339 (8)
F1—B1	1.331 (9)	C11—H11	0.9300
F2—B1	1.357 (9)	C12—C13	1.416 (6)
F3—B1	1.387 (8)	C12—H12	0.9300
F4—B1	1.346 (7)	C13—C14	1.415 (6)
C1—O1	1.370 (5)	C13—C15	1.425 (7)
C1—C2	1.376 (6)	C14—O5	1.311 (5)
C1—C6	1.422 (6)	C15—O6	1.248 (5)
C2—C3	1.397 (7)	C15—H15	0.9300
C2—H2	0.9300	C16—O4	1.426 (6)
C3—C4	1.354 (7)	C16—H16A	0.9600
C3—H3	0.9300	C16—H16B	0.9600
C4—C5	1.423 (6)	C16—H16C	0.9600
C4—H4	0.9300	Cu1—O2	1.888 (3)
C5—C7	1.407 (6)	Cu1—O3	1.929 (3)
C5—C6	1.418 (6)	Cu1—O5	1.889 (3)
C6—O2	1.306 (5)	Cu1—O6	1.936 (3)
C7—O3	1.263 (5)	Cu1—Na1	3.399 (2)
C7—H7	0.9300	Na1—O1	2.615 (3)
C8—O1	1.423 (5)	Na1—O2	2.377 (3)
C8—H8A	0.9600	Na1—O4	2.614 (4)
C8—H8B	0.9600	Na1—O5	2.370 (3)
C8—H8C	0.9600	Na1—F1	2.215 (7)
C9—O4	1.364 (6)	Na1—F3'	2.344 (17)
C9—C10	1.373 (6)	Na1—F4^i^	2.243 (4)
C9—C14	1.415 (6)		
			
B1—F3'—Na1	127.8 (12)	O6—C15—H15	115.7
B1—F1—Na1	133.9 (6)	C13—C15—H15	115.7
B1—F4—Na1^ii^	160.3 (4)	O4—C16—H16A	109.5
F1'—B1—F3'	101 (2)	O4—C16—H16B	109.5
F1'—B1—F2'	92 (2)	H16A—C16—H16B	109.5
F3'—B1—F2'	121.7 (19)	O4—C16—H16C	109.5
F1'—B1—F4	132.8 (15)	H16A—C16—H16C	109.5
F3'—B1—F4	109.5 (10)	H16B—C16—H16C	109.5
F4—B1—F2'	100.8 (15)	O2—Cu1—O5	84.72 (13)
F1—B1—F2	107.9 (7)	O2—Cu1—O3	94.30 (13)
F4—B1—F2	109.6 (6)	O5—Cu1—O3	179.02 (12)
F1—B1—F3	109.8 (7)	O2—Cu1—O6	177.29 (14)
F4—B1—F3	108.3 (5)	O5—Cu1—O6	93.78 (13)
F2—B1—F3	108.9 (6)	O3—Cu1—O6	87.19 (13)
F1—B1—F4	112.4 (7)	O2—Cu1—Na1	42.47 (9)
O1—C1—C2	125.9 (4)	O5—Cu1—Na1	42.27 (9)
O1—C1—C6	113.4 (4)	O3—Cu1—Na1	136.76 (10)
C2—C1—C6	120.7 (5)	O6—Cu1—Na1	135.94 (11)
C1—C2—C3	120.5 (5)	F1—Na1—F4^i^	105.5 (3)
C1—C2—H2	119.7	F1—Na1—F3'	27.6 (4)
C3—C2—H2	119.7	F4^i^—Na1—F3'	88.1 (4)
C4—C3—C2	120.7 (4)	F1—Na1—O5	104.2 (3)
C4—C3—H3	119.6	F4^i^—Na1—O5	126.93 (16)
C2—C3—H3	119.6	F3'—Na1—O5	131.4 (5)
C3—C4—C5	120.4 (5)	F1—Na1—O2	120.2 (3)
C3—C4—H4	119.8	F4^i^—Na1—O2	128.62 (14)
C5—C4—H4	119.8	F3'—Na1—O2	122.4 (5)
C7—C5—C6	122.5 (4)	O5—Na1—O2	64.85 (11)
C7—C5—C4	117.8 (4)	F1—Na1—O4	74.3 (2)
C6—C5—C4	119.7 (4)	F4^i^—Na1—O4	85.33 (15)
O2—C6—C5	124.0 (4)	F3'—Na1—O4	93.7 (6)
O2—C6—C1	118.2 (4)	O5—Na1—O4	62.07 (11)
C5—C6—C1	117.8 (4)	O2—Na1—O4	126.91 (13)
O3—C7—C5	127.8 (4)	F1—Na1—O1	106.6 (2)
O3—C7—H7	116.1	F4^i^—Na1—O1	84.28 (14)
C5—C7—H7	116.1	F3'—Na1—O1	83.9 (6)
O1—C8—H8A	109.5	O5—Na1—O1	126.69 (12)
O1—C8—H8B	109.5	O2—Na1—O1	62.17 (10)
H8A—C8—H8B	109.5	O4—Na1—O1	169.41 (13)
O1—C8—H8C	109.5	F1—Na1—Cu1	116.8 (3)
H8A—C8—H8C	109.5	F4^i^—Na1—Cu1	135.96 (13)
H8B—C8—H8C	109.5	F3'—Na1—Cu1	135.7 (4)
O4—C9—C10	126.1 (5)	O5—Na1—Cu1	32.42 (7)
O4—C9—C14	113.6 (4)	O2—Na1—Cu1	32.44 (7)
C10—C9—C14	120.2 (5)	O4—Na1—Cu1	94.48 (10)
C9—C10—C11	121.4 (5)	O1—Na1—Cu1	94.40 (9)
C9—C10—H10	119.3	C1—O1—C8	117.4 (4)
C11—C10—H10	119.3	C1—O1—Na1	118.6 (2)
C12—C11—C10	119.7 (5)	C8—O1—Na1	124.0 (3)
C12—C11—H11	120.1	C6—O2—Cu1	126.6 (3)
C10—C11—H11	120.1	C6—O2—Na1	127.5 (3)
C11—C12—C13	120.9 (5)	Cu1—O2—Na1	105.09 (12)
C11—C12—H12	119.5	C7—O3—Cu1	124.2 (3)
C13—C12—H12	119.5	C9—O4—C16	118.2 (4)
C14—C13—C12	120.1 (5)	C9—O4—Na1	118.6 (3)
C14—C13—C15	121.2 (4)	C16—O4—Na1	122.6 (3)
C12—C13—C15	118.7 (5)	C14—O5—Cu1	127.0 (3)
O5—C14—C9	117.9 (4)	C14—O5—Na1	127.7 (3)
O5—C14—C13	124.6 (4)	Cu1—O5—Na1	105.31 (13)
C9—C14—C13	117.5 (4)	C15—O6—Cu1	124.6 (3)
O6—C15—C13	128.5 (5)		

Symmetry codes: (i) *x*, −*y*+3/2, *z*+1/2; (ii) *x*, −*y*+3/2, *z*−1/2.
